# A cluster randomised trial of a school-based intervention to prevent decline in adolescent physical activity levels: study protocol for the ‘Physical Activity 4 Everyone’ trial

**DOI:** 10.1186/1471-2458-13-57

**Published:** 2013-01-22

**Authors:** Rachel Sutherland, Elizabeth Campbell, David R Lubans, Philip J Morgan, Anthony D Okely, Nicole Nathan, Luke Wolfenden, Jannah Jones, Lynda Davies, Karen Gillham, John Wiggers

**Affiliations:** 1 Hunter New England Population Health, Locked Bag 10, Wallsend, NSW, 2287, Australia; 2 School of Medicine and Public Health, University of Newcastle, Newcastle, 2308, Australia; 3 Hunter Medical Research Institute, Newcastle, NSW, 2300, Australia; 4 Priority Research Centre in Physical Activity and Nutrition, School of Education, University of Newcastle, Newcastle, Australia; 5 Interdisciplinary Educational Research Institute and Faculty of Education, University of Wollongong, Wollongong, Australia

**Keywords:** Physical activity, Adolescents, School, Randomized controlled trial

## Abstract

**Background:**

Adolescence is an established period of physical activity decline. Multi-component school-based interventions have the potential to slow the decline in adolescents’ physical activity; however, few interventions have been conducted in schools located in low-income or disadvantaged communities. This study aims to assess the effectiveness of a multi-component school-based intervention in reducing the decline in physical activity among students attending secondary schools located in disadvantaged communities.

**Methods/Design:**

The cluster randomised trial will be conducted with 10 secondary schools located in selected regions of New South Wales, Australia. The schools will be selected from areas that have a level of socio-economic status that is below the state average. Five schools will be allocated to receive an intervention based on the Health Promoting Schools framework, and will be supported by a part-time physical activity consultant placed in intervention schools who will implement a range of intervention adoption strategies. Study measures will be taken at baseline when students are in Year 7 (12–13 years) and again after 12- and 24-months. The primary outcome, minutes of moderate- to-vigorous- intensity physical activity per day and percentage of time in moderate- to vigorous-intensity physical activity (MVPA), will be objectively assessed using accelerometers (Actigraph GT3x+). Group allocation and intervention delivery will commence after baseline data collection. The intervention will continue during school terms through to 24-month follow-up.

**Discussion:**

The study will provide evidence regarding the effectiveness of a multi-component school-based intervention that includes an in-school physical activity consultant targeting the physical activity levels of adolescents in disadvantaged Australian secondary schools.

**Trial registration:**

Australian New Zealand Clinical Trials Registry ACTRN12612000382875.

## Background

Being physically active can prevent numerous chronic diseases including coronary heart disease, obesity, some types of cancers such as colorectal and breast cancers, and improve muscle strength and fitness and aspects of mental health [1,2]. Despite such benefits, population surveys from the United States of America and the United Kingdom have found that only 15.3 percent of 13–18 year olds [3] in the United States of America and as few as 0–7 percent of 11–15 year olds [4] from the United Kingdom being physically active to a sufficient level to improve health. Similarly, the proportion of Australians adolescents aged 13 to 17 years that meet the recommended amount of physical activity is around 15 percent [5].

The transition into adolescence is a recognised period of physical activity decline. Research suggests that moderate to vigorous physical activity drops by up to seven percent per year between the ages of 9 to 15 years, so that by age 15 the majority of adolescents no longer meet the recommended daily amount of activity [6,7]. In addition, the physical activity decline associated with adolescence is steeper among youth from disadvantaged or low income communities [8,9]. Reducing this decline is an important health priority as inactivity tends to track into adulthood [10].

Schools are a key setting for the promotion of physical activity as they have existing curricula, infrastructure, policies and resources to promote physical activity [11] and are also able to reach those from all backgrounds [12]. The effectiveness of multi-component school based physical activity interventions, particularly those that include links to families and communities, has consistently been demonstrated in reviews [13-19]. However, recent reviews of school based physical activity interventions, have identified only three studies focusing on low income groups [13,20-22] each targeting children of primary school age (i.e. aged 6 to 12 years) rather than adolescents.

Schools located in disadvantaged communities face a number of challenges in implementing whole of school physical activity programs, including student, teacher and parent disengagement and high staff turnover [23-26]. In addition, Australian research indicates students from lower socio-economic or disadvantaged backgrounds face barriers in physical activity participation including lack of parental support, cost of school sports, time available for school sport and choice and variety of physical activities offered at school [27,28]. A review of the effectiveness of physical activity interventions in disadvantaged groups, although not specific to schools, concluded that interventions underpinned by a theoretical framework were more likely to be successful, and suggested the importance of focussing on a range of areas including social and professional supports and increasing the length of the intervention period [18]. The Health Promoting Schools (HPS) framework [29] has an emphasis on intervention across a range of areas including school curriculum, school environment and ethos, and partnerships with community and parents. In order to address the challenges of intervening within disadvantaged schools, in addition to the introduction of health strategies, the explicit incorporation of strategies to support intervention adoption within schools has been suggested to be important [24,30,31]. Such strategies can include teacher professional learning, on-going teacher support, availability of credible leadership and opinion leaders, provision of resources and prompts, and monitoring and feedback of intervention adoption [24,30,31].

Given the lack of multi-component intervention studies that target physical activity levels among adolescents from lower income or disadvantaged groups, the aim of this study is to determine whether a multi-component physical activity intervention implemented in disadvantaged secondary schools can reduce the decline in physical activity associated with adolescence.

## Methods/Design

### Study design

**Figure 1:**
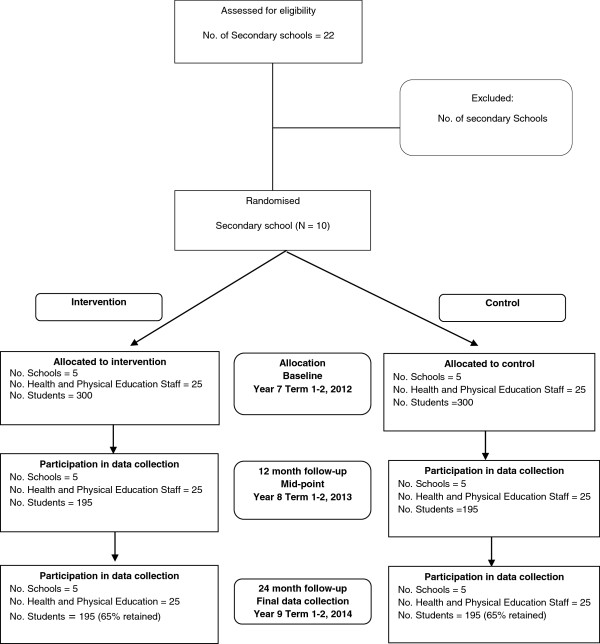
CONSORT flowchart describing progress of participants through the study.

This study will employ a cluster randomised controlled design (Figure 1).

The research will be conducted and reported in accordance with the requirements of the Consolidated Standards of Reporting Trials (CONSORT) Statement [32,33]. A randomly selected sample of disadvantaged secondary schools in the study region will be invited to participate. The schools will be randomly allocated to receive a multi-component intervention to be implemented during school terms and to commence after baseline data collection, or to a control group. Data will be collected from students at baseline (when students are in their first year of high school, aged 12–13 years), and from the same cohort of students after 12-months (midpoint) and 24-months post baseline data collection. The primary outcome will be minutes per day and percentage of time in moderate- to vigorous-intensity physical activity (MVPA) at 24-month follow-up.

### Setting

The study will be conducted in the Hunter, Central Coast and Mid North Coast regions of the state of New South Wales, Australia. These regions encompass major city and regional areas (ARIA) [34]. The regions have lower indices of socio-economic status than New South Wales [35] and a population of approximately 64,188 children aged between 12 and 15 years (17.6%) [35].

### Sample/ Participants

#### Secondary schools

Secondary schools in New South Wales cater for students aged from about 12 (Year 7) to 18 (Year 12) years old. Students are required to undertake 300 hours of Health and Physical Education each year, from Year 7 to Year 10 [36,37]. Students also have opportunities to engage in physical activity through school sport (averaging 2 hours per week) [38]. Physical Education is taught by qualified Health and Physical Education teachers.

Of the secondary schools within the study region, those that meet the following criteria will be eligible to participate in the study: Government and Catholic schools; schools with postcodes ranked in the bottom 50% of New South Wales postcodes based on the Socio-Economic Indexes For Australia (SEIFA) [39]; have between 120–200 Year 7 students (to meet sample size requirements); and are not participating in other major physical activity or health intervention studies. Ten schools will be recruited.

#### Students

All Year 7 students in participating schools will be eligible to participate in the study measurement. Classes catering for students with severe physical and mental disabilities will be excluded.

### Recruitment procedures

#### Schools

Prior to recruitment, the study will be promoted to school sector Regional Directors within the NSW Department of Education and Communities (DEC) and the relevant Catholic school Dioceses to gain their support. A random number function in Microsoft Excel will be used to determine the order in which the eligible secondary schools are approached to participate. Invitations to participate will be sent to the 10 randomly selected schools. If a selected school declines, an additional letter will be sent to the next eligible school on the list, until 10 schools accept the invitation to participate.

A letter will be sent to selected schools, detailing the study and inviting participation. Approximately two weeks after the invitation letter is sent to the school, the Principal will be contacted by phone by a member of the research team. A face-to-face meeting will be requested with both the Principal and the Head Health and Physical Education teacher to outline the requirements of the study and request consent.

#### Random allocation of schools

Eligible schools will be classified into two strata based on geographic location (regional or major city) [34]. Research indicates that location may contribute to the varying physical activity levels of adolescents [40]. Four consenting schools will be obtained from the major city strata and six from the regional school strata. Participating schools will be randomly allocated using block randomization (1:1 ratio) to the intervention or control condition using a computerized random number function. Randomization will be undertaken by a statistician not involved in contacting schools or in the study intervention or assessment and will occur after baseline data collection to reduce participation bias from students, teachers and researchers. The school Principal will receive a letter from the study team indicating to which group the school has been allocated. Data collectors will be blinded at baseline and where possible at 12 and 24-months data collection.

#### Students

All Year 7 students in the participating schools will be provided an information package that will contain a letter outlining the study and a consent form for parents asking for consent for their child to participate in the study data collection. Parents will be provided with a telephone number where they can leave a message if they do not want to be prompted about consent or do not want their child to participate in the measurement component of the intervention. Two weeks following distribution of the information package, parents who have not returned a consent form or left a message indicating they do not wish to be contacted, will be telephoned by staff employed through the education sector and asked if their child can participate in study measurement. A replacement consent form will be sent to parents providing verbal consent.

A number of strategies that have been used successfully in similar research will be adopted in an to effort maximise parent and student consent. These include having a designated recruitment co-ordinator, promoting the research prior to requests to participate, disseminating materials to maximise parent engagement, and issuing reminders to parents using a variety of methods including phone calls and letters [28,38,41].

### Physical activity intervention

#### Theoretical framework and physical activity content

Consistent with recommendations from reviews to maximise the effectiveness of school-based physical activity studies, the intervention has been guided by social cognitive [42] and social-ecological theories [43] and will be implemented using the World Health Organisation’s (WHO) Health Promoting Schools framework. This framework includes strategies that address the school curriculum, school environment and community [14,29,44-46].

**Figure 2:**
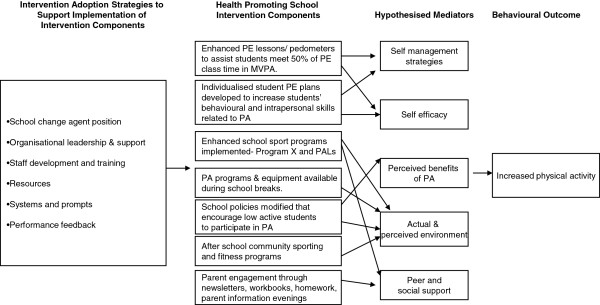
**Intervention Adoption Strategies to Support Implementation of Intervention Components.** Health Promoting School Intervention Components. Hypothesised Mediators. Behavioural Outcome.

Figure 2 shows the seven physical activity intervention strategies that schools will be facilitated to implement, and the strategies that will be used to increase the extent of intervention adoption. A further description of the physical activity intervention strategies within the Health Promoting Schools framework domains is as follows.

Formal curriculum

1. Implementation of teaching strategies to maximise student activity levels within Physical Education classes. Schools will aim to meet 50% of Physical Education class time in MVPA for their students, a standard recommended by the US Centres for Disease Control (CDC) and Prevention [47]. Health and Physical Education teachers will receive training and resources to assist in maximising moderate- vigorous physical activity, including workshop style sessions that will incorporate a facilitated process for reflection on levels of MVPA in lessons and changing teaching practices to enhance levels of activity. In addition to this training, regular pedometer-based lessons and curriculum material will be introduced to assist teachers [48-50].

2. Development and monitoring of annual individual student physical activity plans in health and physical activity that include: long- and short-term personal goals for improving or maintaining regular physical activity; specific actions and timelines to achieve those goals; fitness assessments; methods to be used to record actions and assess progress; and rewards for achieving goals [51]. Health and Physical Education teachers will be responsible for co-ordinating the development and monitoring of individual physical activity plans. Consistent with the CDC guidelines, students will be encouraged to review their physical activity plans and modify the content regularly [11]. Students will also be given small incentives when their personal goals have been met (such as balls, wrist bands, drink bottles).

3. Implementation of enhanced school sport programs for all students. All students will be scheduled to participate in age appropriate 10-week programs during school sport while they are in Years 7, 8 or 9. The school sports programs will be based on *Program X* and Physical Activity Leaders (*PALs*), both of which have been shown to be efficacious in adolescents [48,52]. These single sex programs include; health-related fitness activities, pedometers for self-monitoring, lunch-time activities, information for parents and interactive seminars [38,48,52]. The programs have been designed to meet the needs of low-active students, and are known to be acceptable and appropriate for such students and improve psychological outcomes [53]. The program content is relevant to all students, and year-wide implementation as a sport option will ensure no student is stigmatised.

School ethos and environment

4. Modification of school policies that encourage low-active students to be more physically active [54]. The policies within each intervention school will be reviewed with the aim of establishing policies that enhance physical activity, and modifying existing policies that may be inhibiting activity. School policies that promote single sex Physical Education classes, modified Physical Education uniforms for girls, mandatory Physical Education and sport, provision of equipment and staff supervision in breaks have been shown to enhance physical activity [55].

5. Implementation of daily physical activity programs for students during school breaks including increasing the availability of facilities and equipment. Intervention schools will be offered equipment and supervised activities in recess and lunch breaks. Supervised activities have been shown to increase the participation of students in recess and lunchtime activities [56]. Through participation in the enhanced sport programs (ie. Program X and PALs), the intervention aims to train students to lead the recess and lunchtime activities over the course of the intervention.

Partnerships and Services

6. Implementation of accessible after-school physical activity programs through linkages with community sporting groups and/or organisations from the fitness industry [54,57]. Links will be established between the school and the broader community to enhance the physical activity opportunities available to students outside of school hours. The types of links established will be based on the criteria used in other physical activity interventions [38,58].

7. Parent engagement: Strategies will encourage parents to; increase their adolescent’s physical activity, be active with their adolescent at home and in the community [59,60]. Regular information will be sent to parents via existing schools newsletters, school website and program newsletters to support the activities occurring within the school. The materials will inform parents of school-based strategies, promote newly established community links and provide ideas to support physical activity outside of school hours. The newsletters will also aim to provide information on physical activity recommendations and strategies to encourage participation, including parent role modelling.

### Intervention adoption strategies

The lack of explicit intervention adoption strategies has been highlighted as a limitation of school based physical activity interventions [13]. To increase the extent of school adoption of the intervention, seven strategies will be used to support implementation of the physical activity components. These strategies are based on literature shown to facilitate the adoption of school based interventions, change service delivery practices of organisations and build capacity of an organization [25,30,31,61-64]. These include:

1. *Change agent position* (*inschool physical activity consultant*): A Health and Physical Education teacher will be located within each school for one day per week over the intervention period to support the planning, and implementation of the program under the guidance of the school Principal and the Head Health and Physical Education teacher. This is consistent with previous research showing that location of a physical activity expert within a school can increase the amount of MVPA in Physical Education lessons [57].

2. *Establishing leadership and support*: A committee will lead and oversee the implementation of the intervention within each school. This role could be taken on by an existing school committee or a new school committee could be established with representatives from the school executive, health and Physical Education staff, staff from other key learning areas, students, parents and community. The committee, with the guidance of the in-school consultant will develop an intervention implementation plan. Meetings will be suggested to occur quarterly. Schools will also be asked to nominate a school co-ordinator whom the in-school consultant can work closely with for the duration of the project with the aim of handing over to this person when the research trial has been completed. Presentations will be given to all school staff in addition to the Health and Physical Education teachers and parents. These presentations will provide a means of gaining support for the project, providing input into the implementation and also inform the school community of progress. School Principals and Health and Physical Education head teachers will be encouraged to provide leadership via raising the program at staff meetings, attending the school committee meetings and approving and implementing supportive policy changes.

3. *Staff Training*: Training from credible professionals has been shown to be an effective implementation support strategy [18,61]. Teachers will be provided with training in the physical activity components relevant to their role in implementation. Health and Physical Education teachers will specifically be trained to deliver Physical Education lessons that increase students MVPA. Training and tips will be regularly provided by the in-school physical activity consultant and further professional development sessions will be held twice a year (4 sessions in total over the intervention). The training will be a series of practical learning workshops designed to foster skill development where schools can jointly share their experiences in implementing the strategies, rather than a didactic lecture style format. Teachers from across the whole school will also be invited and trained to deliver the enhanced school sports programs [48,52].

4. *Resources*: Schools will receive an intervention manual including material to implement the Physical Education lesson strategies, enhanced sports and physical activity programs during school breaks, and material on other strategies. Schools will also receive physical activity equipment such as elastic tubing resistance devices, pedometers, active electronic games consoles, skipping and boxing equipment. In addition to the resource kit provided to schools, additional small promotional incentives such as shirts and lanyards will be provided to teachers upon the introduction of curriculum based strategies. Small promotional incentives (balls, wrist bands, water bottles etc.) will also be given to students upon reaching their personal physical activity goals, for participating in recess and lunchtime activity and completing the enhanced school sports programs.

5. *Prompts*: The physical activity consultant will provide prompts such as emails, reminders in meetings and markings on calendars to teaching staff to undertake intervention strategies. The physical activity consultant will also work with schools to identify ways to build prompts into school communication processes and documents such as electronic calendar reminders and agenda items in meetings.

6. *Intervention adoption performance feedback*: Principals and Head Health and Physical Education teachers at each school will be given feedback on progress on each physical activity intervention strategy against agreed standards at the end of each school term (quarterly). The feedback reports will also include suggestions and offer support on how to improve performance.

### Control schools

Control schools will participate in the measurement components of the study only. Control schools will be offered one day of teacher relief funding at each data collection point (baseline, mid-point and follow-up) to reimburse the school for their time in assisting with data collection. Control schools will also be offered the physical activity equipment pack, all developed intervention materials and the results of the study at the end of the intervention period.

### Data collection procedures and measures

All data will be collected at three time points: baseline; 12- and 24-months. Students will wear an accelerometer to record physical activity levels, undertake an on-line survey, and have anthropometric measures taken. The accelerometers and instructions for use will be distributed to students at school within class time, at the same time as students complete the online survey and have anthropometric measures taken.

#### Outcome measure- physical activity levels

The primary outcome will be student physical activity defined as mean minutes of MVPA. Percentage of time spent in MVPA will also be calculated to adjust for individual accelerometer wear time.

Objectively measured physical activity data will be collected via accelerometers (Actigraph GT3X+ and GT3X model). Accelerometry provides an objective, valid and reliable way of measuring physical activity in young people [65-67]. Students will be asked by trained research assistants to wear the accelerometers during waking hours for seven consecutive days. The accelerometers will be attached to an elastic belt and worn over the right hip. Raw data will be collected and stored in 15 second epochs. Student data will be analysed if accelerometers are worn for ≥ 600 minutes on ≥ 3 days [68]. The Evenson cut-points will be used to categorize different intensities of physical activity [69]. Students will also be asked to keep activity monitoring logs for the seven-day period when the accelerometers were being worn. To improve compliance, students will be sent a text message each morning reminding them to wear the accelerometer [28,38]. Student and/ or parent mobile phone numbers will be requested via the consent form.

#### Student characteristics

An online survey, which will take approximately 30 minutes, will be undertaken to assess student socio-demographic characteristics (age, gender, Aboriginal or Torres Strait Islander status and postcode of residence), self-reported physical activity [70], physical activity mediators [28,71].

#### Anthropometric data

Anthropometric data including height and weight will be collected. Research assistants will be trained in measuring height, weight (used to calculate body mass index; BMI) and waist circumference using the International Society for the Advancement of Kinanthropometry (ISAK) procedures [72]. Weight will be measured in light clothing without shoes using a portable digital scale (Model no. UC-321PC, A&D Company Ltd, Tokyo Japan) to the nearest 0.1 kg. Height will be recorded to the nearest 0.1 cm using a portable stadiometer (Model no. PE087, Mentone Educational Centre, Australia). Waist measurement will be taken as the narrowest point between the inferior rib border and the iliac crest. Using a flexible but inelastic tape measure, the waist measure will be recorded to the nearest 0.1 cm. Two recordings will be taken and then the average will be used. The physical assessments will be conducted in a sensitive manner, with student measurements taken behind a privacy screen. Body mass index (BMI) will be calculated as weight/height squared (kg/m^2^). Weight status will be determined using International Obesity Taskforce definitions [73].

#### School outcomes/Process measures

Data regarding school policies and practices that enhance student physical activity will be collected at baseline and follow-ups via a school environment survey completed by the Head Health and Physical Education teacher at intervention and control schools. Based on existing surveys [74], the survey will focus on school policies and practices that enhance student physical activity including questions relating to school equipment and facilities, recess and lunch activities and rewards and punishments related to physical activity.

In addition, observational assessments of randomly selected Physical Education classes in both intervention and control schools will be undertaken at each time point to measure physical activity levels, lessons context and teacher interactions [75]. The observational tool, SOFIT (System for Observing Fitness Instruction Time), has been used in similar studies to assess physical activity levels in Physical Education classes [57]. Trained research assistants will conduct the assessments on the same weeks that student data collection is occurring.

Data will be collected by project staff throughout the intervention period to assess the extent of intervention adoption and implementation fidelity in intervention schools.

### Sample size

Based on an estimate of 50% of Year 7 students consenting and providing usable accelerometer data, each school should yield at least 60 students (based on at least 120 Year 7 students). This will provide at least 300 students per group. Based on an estimated 65% of the cohort providing usable data at follow-up, there will be at least 195 students per group at 24-month follow-up. Previous studies have been used to estimate the standard deviation of mean daily minutes MVPA per group (17.1) [28] and the Intra Class Correlation coefficient (ICC) (0.01) [76]. After adjustment for the design effect of 1.38, it is estimated the effective sample size will be at least 141 students per group. With this sample size, with 80% power and an alpha level of 0.05 the study will be able to detect a difference in the mean daily MVPA between experimental and control students of +/− 5.73 minutes at follow-up.

### Statistical analysis – primary outcome

Analyses using cluster-level summaries are more robust than analyses based on individual-level data when there are less than 15 clusters per treatment arm [77]. Therefore the primary outcome for this study will be analysed by calculating the change in the mean number of minutes of moderate to vigorous physical activity within each school and then comparing the school-level means of the intervention group with the school-level means of the control group using a two sample t-test. The main analysis will be conducted with all available data using the intention to treat principle and sensitivity analyses conducted under various assumptions about the missing data mechanism [78]. Per protocol analyses will also be performed where appropriate.

## Discussion

Despite a recent increase in school-based physical activity interventions, few have targeted adolescents living in low-income communities [9,14,79]. To the research team’s knowledge, this is the first study targeting both boys and girls in disadvantaged communities that has used a whole of school approach to physical activity promotion in this cohort and uses an objective physical activity measure. This approach combines strategies shown to be effective in increasing or maintaining physical activity for both boys and girls, and also for enhancing activity for students classified as low-active. The intervention also incorporates strategies known to facilitate intervention adoption, including the use of an in-school physical activity consultant to be placed in intervention schools for the intervention period. This support strategy is designed to overcome a number of barriers reported to inhibit school based interventions such as providing adequate training and resources, enhancing communication, providing ongoing and regular support to teachers implementing the intervention and gaining leadership and support from the school executive, parents and the community [24,80,81]. The study has also been designed with a longer follow-up period.

The study can contribute to the literature by identifying whether the intervention approach can increase physical activity or reduce the decline in physical activity among adolescents living in low income communities.

## Abbreviations

PA: Physical activity; PA4E1: Physical activity for everyone; CATI: Computer assisted telephone interview; HPS: Health promoting schools; MVPA: Moderate-to-vigorous physical activity; HPE: Health and physical education.

## Competing interests

The authors declare that there are no competing interests.

## Authors’ contributions

JW, PJM, DRL, LC, LW, KG obtained funding for the research. All authors contributed to developing the intervention and data collection protocols and materials, and reviewing, editing, and approving the final version of the paper. RS, JJ, NN and LD organized and conducted all of the assessments. All authors accept full responsibility for, and have read and approved the final manuscript.

## Pre-publication history

The pre-publication history for this paper can be accessed here:

http://www.biomedcentral.com/1471-2458/13/57/prepub
